# Bedside Catheter Hematoma Evacuation in Vitamin K Antagonist-Related Intracerebral Hemorrhage: A Safe and Feasible Approach

**DOI:** 10.3389/fneur.2020.00807

**Published:** 2020-08-14

**Authors:** Bastian Volbers, Wolf-Dirk Niesen, Samuel Amiri-Soltani, Dimitre Staykov, Mukesch Johannes Shah, Stefan Lang, Hannes Lücking, Joji B. Kuramatsu, Hagen B. Huttner, Stefan Schwab, Jürgen Bardutzky

**Affiliations:** ^1^Department of Neurology, University Medical Center Erlangen-Nuremberg, Erlangen, Germany; ^2^Department of Neurology, University of Freiburg, Freiburg im Breisgau, Germany; ^3^Department of Neurology, Hospital of the Brothers of St. John, Eisenstadt, Austria; ^4^Department of Neurosurgery, University Medical Center Freiburg, Freiburg im Breisgau, Germany; ^5^Department of Neuroradiology, University Medical Center Erlangen-Nuremberg, Erlangen, Germany

**Keywords:** minimally invasive surgery, intracerebral hemorrhage, vitamin K antagonist, peri-hemorrhagic edema, midline shift

## Abstract

**Background and Purpose:** Although outcome in intracerebral hemorrhage (ICH) patients is generally not improved by surgical intervention, the use of minimally invasive surgery (MIS) has shown promising results. However, vitamin K antagonist (VKA)-related ICH patients are underrepresented in surgical treatment trials. We therefore assessed the safety and efficacy of a bedside MIS approach including local application of urokinase in VKA-related ICH.

**Methods:** Patients with a VKA-related ICH > 20 ml who received bedside hematoma evacuation treatment (*n* = 21) at the University Medical Center Freiburg were retrospectively included for analysis and compared to a historical control group (*n* = 35) selected from an institutional database (University Medical Center Erlangen) according to identical inclusion criteria. Propensity score matching was performed to obtain comparable cohorts. The evolution of hematoma and peri-hemorrhagic edema (PHE) volumes, midline shift, and the occurrence of adverse events were analyzed. Furthermore, we assessed the modified Rankin Scale and NIHSS scores recorded at discharge.

**Results:** Propensity score matching resulted in 16 patients per group with well-balanced characteristics. Median ICH volume at admission was 45.7 (IQR: 24.2–56.7) ml in the control group and 48.4 (IQR: 28.7–59.6) ml in the treatment group (*p* = 0.327). ICH volume at day 7 was less pronounced in the treatment group [MIS: 23.2 ml (IQR: 15.8–32.3) vs. control: 43.2 ml (IQR: 27.5–52.4); *p* = 0.013], as was the increase in midline shift up to day 7 [MIS: −3.75 mM (IQR: −4.25 to −2) vs. control: 1 mM (IQR: 0–2); *p* < 0.001]. No group differences were observed in PHE volume on day 7 [MIS: 42.4 ml (IQR: 25.0–72.3) vs. control: 31.0 ml (IQR: 18.8–53.8); *p* = 0.274] or mRS at discharge [MIS: 5 (IQR: 4–5) and 5 (IQR: 4–5); *p* = 0.949]. No hematoma expansion was observed. The catheter had to be replaced in 1 patient (6%).

**Conclusions:** Bedside catheter-based hematoma evacuation followed by local thrombolysis with urokinase appears to be feasible and safe in cases of large VKA-related ICH. Further studies that assess the functional outcome associated with this technique are warranted.

**Clinical Trial Registration:** DRKS00007908 (German Clinical Trial Register; www.drks.de)

## Introduction

Many attempts have been made at improving the poor functional outcome in intracerebral hemorrhage (ICH) patients. One of the major pathophysiological factors related to a poor outcome has been identified as increased hematoma volume, which either causes mechanical disruption or space-occupying mass effects, or can induce the activation of secondary detrimental pathways that lead to peri-hemorrhagic edema, inflammation, and apoptosis (1, 2). Thus, primary clot reduction theoretically represents a promising treatment target. However, several clinical (3, 4) trials as well as meta-analyses (5, 6) have so far failed to demonstrate a clear clinical benefit of clot removal via craniotomy compared to best medical treatment in ICH patients. Furthermore, only a subset of patients with superficial ICH might be able to benefit from this procedure (2). Given that very few vitamin K antagonist (VKA)-related ICH patients have been included in the aforementioned clinical trials, the data regarding VKA-associated hemorrhage is even more limited.

Minimally invasive surgery (MIS) is a potential alternative approach that yields promising results, even in patients with deep ICH, provided that a sufficient reduction in hematoma size is achieved. Since the late 1980s, several publications have reported an improvement in short- and long-term mortality in ICH patients treated with endoscopic evacuation compared to those who received standard medical management (7, 8), while a retrospective case series also showed an association between endoscopic evacuation and improved functional outcome (9). However, several clinical trials including the multi-centric, prospective Minimally Invasive Surgery Plus Rt-PA for ICH Evacuation III (MISTIE III) trial were unable to detect a functional benefit in the intention-to-treat cohort (2, 10). Peri-hemorrhagic edema was shown to be reduced in MIS-treated patients in the MISTIE II-Trial (11). Nonetheless, these MIS-trials (10–15) only included very few patients with VKA-related ICH.

Patients with VKA-related ICH are at risk of a higher secondary hematoma expansion rate, a larger hematoma volume, and hence a poor functional outcome (16, 17). Although the available findings on the MIS technique are encouraging, no sufficient data on efficacy and safety of existing MIS technologies (18) are available for patients in VKA-related hemorrhage (2). The aim of the present retrospective case–control study was therefore to generate novel data on the safety and efficacy of a minimally invasive catheter evacuation technique in patients with VKA-related deep and lobar ICH. In this approach, hematoma reduction is achieved by free-hand, bedside catheter placement with aspiration, followed by catheter irrigation with urokinase (19, 20).

## Methods

### Patient Selection

Patients with VKA-related supratentorial ICH > 20 ml [International Normalized Ratio (INR) on admission ≥1.8] from two distinct hospitals were included in this retrospective study.

In the first hospital, patients were treated by bedside catheter aspiration of the hematoma, followed by fibrinolytic therapy after immediate anticoagulant reversal (MIS group). In the second hospital, patients with VKA-related ICH received the maximum medical treatment and served as controls (control group). The study was approved by the local institutional ethics committee (University of Freiburg, No. 161/19; University of Erlangen-Nuremberg Re.-No. 4481).

### Control Group

A historical control group was selected from a prospectively organized institutional database (Department of Neurology, University of Erlangen-Nuremberg). The inclusion criteria were a VKA-related supratentorial ICH with a hematoma volume >20 ml admitted between 2010 and 2017; reduced level of consciousness; INR <1.3 after administration of 4-factor prothrombin complex concentrate (PCC); age ≥18 years; and at least 2 CT scans up to 7 days after admission. The exclusion criteria included the early withdrawal of treatment (within 24 h of admission), or an ICH due to vascular malformation detected in CT angiography.

In the control group, a stability CT on admission and further CT scans during the hospital stay were performed according to institutional protocols in cases of clinical deterioration, or at the discretion of the treating physician.

### Treatment Group (MIS Group)

Patients admitted to the Department of Neurology, University Hospital Freiburg, between 2011 and 2017 were identified from a prospectively organized institutional database. Patients were treated with MIS when the following criteria were fulfilled: (I) VKA-related supratentorial ICH; (II) hematoma volume > 20 ml, (III) reduced level of consciousness due to ICH (somnolence at the least); (IV) INR <1.3 after administration of PCC, (V) platelet count >100,000/μl; (VI) exclusion of underlying vascular malformation in CT angiography. An exclusion criterion was the early withdrawal of treatment (within 24 h of admission).

### MIS Procedure

In the 3D mode of the J-Vision Picture Archiving and Communication System (PACS) (TIANI Medgraph, Bonn Germany), the extent of hemorrhage in axial, sagittal, and transversal views was used to determine the most favorable catheter trajectory for avoiding critical intracerebral structures. Using the CT scan, the location for the burr hole was selected, and the respective distances from the midline and the skin level to the desired location of the tip of the catheter were determined. Patients were placed in the supine position and the midline was calculated by measuring the circumference from one external auditory meatus to the other. The coordinates were transferred to the patient's head, a 6 × 6 cm area of hair was shaved, and the skin was sanitized. Five milliliters of scandicain was used to infiltrate the skin and periosteum, and i.v. propofol was used for conscious sedation (median: 110 mg; range: 70–190 mg). None of the patients required intubation and mechanical ventilation during the procedure.

A 3.5-mm burr hole was made using a hand drill, and a scaled external ventricular catheter (Spiegelberg, Hamburg, Germany) was inserted at the calculated angle and depth.

An anterior trajectory pathway was generally used for cases of deep ICH that occupied the anterior basal ganglia, with an entry point at the forehead; a posterior trajectory path was used for cases of deep-seated ICH that occupied the posterior basal ganglia or thalamus, with an entry point at the posterior parietal–occipital area. For lobar ICH cases, the entry point of the trajectory was the superficial area closest to the hematoma. The catheter was deemed to be in a good position when placed along the entire longitudinal axis with at least two-thirds of longitudinal length of the hematoma and the fenestrated segment in the center of the clot.

**Figure 1:**
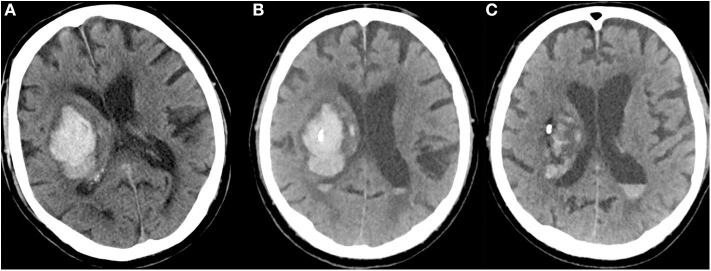
**(A)** CT of a 79-year-old man with a right-sided, VKA-related deep ICH at admission (NIHSS = 14; INR = 3.0; hematoma volume: 58 ml). **(B)** CT after bedside catheter placement and aspiration of 11 ml of blood shows that the catheter tip is well positioned in the core of the hematoma (after administration of 4000 PCC, INR = 1.2). **(C)** After application of urokinase (5000 IE every 6 h) for four consecutive days, the hematoma volume had decreased to 21 ml (NIHSS = 10).

Mild aspiration using a syringe helped to drain blood immediately, the catheter was attached to the skin, and a sterile drainage system was attached. A control CT was performed to verify catheter position and residual hematoma volume after aspiration. At approximately 3–5 h after catheter placement, urokinase (5000 IE, 1 ml) was injected and the system was flushed with 2 ml of 0.9% saline. The drain was clamped for 30 min to ensure an adequate exposure time and then re-opened to allow for gravitational drainage of the lysed clot. Lysis (5000 IE urokinase, 1 ml) was repeated every 6 h, either until the hematoma volume decreased to <50% of the initial volume, or for a maximum of 4 days. Cranial CT scans were generally carried out on day 2 or 3 to verify intrahematomal catheter position and evaluate the remaining hematoma volume; this was repeated between days 4–7. See Figure 1 for an exemplary case with MIS.

### Medical Treatment

All patients in the control and MIS groups received medical treatment according to institutional procedures and the European Stroke Initiative guidelines for the monitoring and treatment of ICH (21, 22). This included immediate anticoagulant reversal (INR target <1.3) using PCC, and immediate lowering of systolic blood pressure to <140 mmHg.

According to the institutional protocols in both centers, open surgical evacuation was not recommended within the first 24 h of VKA-related ICH. Open surgery may have only been considered a lifesaving option in patients with clinical and radiological signs of incipient herniation, due to the mass effect of ICH.

### Neuroimaging and Assessment of Peri-Hemorrhagic Edema (PHE)/ICH Volume and Midline Shift

Neuroimaging in both hospitals was performed on a fourth-generation computed tomography (CT) scanner (Somatom 64, Somatom Definition AS+, Siemens Healthcare, Erlangen). Each CT scan consisted of 10–12 slices at a thickness of 4.8 mm for the skull base; 10–12 slices at a thickness of 7.2 mm for the cerebrum (Somatom 64); 22–25 slices at a thickness of 4.8 mm for the entire brain (Somatom AS+); or a multi-slice spiral CT data set. CT images were acquired in the orbito-meatal plane. For analysis, the absolute ICH and PHE volumes were obtained using a validated semi-automatic volumetric algorithm as previously described, with a high level of inter/intra-rater reliability. A threshold of 5–33 Hounsfield Units was used for calculating PHE volume. All measurements were performed by B.V (23). Midline shift was assessed by measuring the distance (mm) between the third ventricle and a designated midline drawn between the anterior and posterior attachments of the falx to the inner table of the skull. For better temporal comparison, the different time points of the CT scans were merged into time clusters (days 1, 2–3, and 4–7). All CT scans were included for analysis, especially for the purposes of assessing hematoma expansion. Hematoma volume, PHE volume, and midline shift were also recorded.

### Endpoints

The evolution of hematoma and PHE volumes as well as the midline shift were analyzed as primary outcome variables. For the secondary outcome variable, we assessed functional outcome according to the modified Rankin Scale (mRS), the National Institutes of Health Stroke Scale (NIHSS), and the Glasgow Coma Scale (GCS), both at discharge from hospital and in cases of in-hospital mortality. As safety parameters, any growth in hematoma volume observed by CT, either after drainage placement or during and after local lysis, was assessed, as was symptomatic hematoma expansion [worsening of NIHSS score (>4) associated with hematoma expansion] and the occurrence of ventriculitis. Ventriculitis was diagnosed according to the criteria described by Lozier et al. (24). Routine microbiological testing of the catheter tip was not performed upon its removal. In MIS patients, secondary hematoma expansion was both generally and specifically assessed after MIS was performed, and then generally compared to control patients.

The Modified Rankin Scale, GCS, and NIHSS scores at discharge were obtained from medical records. If no score was recorded at discharge, a certified rater (B.V./S.A-S.) evaluated each patient based on the available clinical notes.

### Statistics

Statistical analyses were performed using the IBM® SPSS® Statistics 21 software package (IBM Corporation, Armonk, NY) and R 2.12.0 (http://www.r-project.org). The significance level was set at α = 0.05 and statistical tests were two-sided. Any missing data relating to basic characteristics, neuroimaging, or outcome led to exclusion of the patient concerned. Twenty-one patients were allocated to a drainage insertion and 35 patients were allocated to the control group based on inclusion criteria. Since the initial hematoma volume differed significantly between groups, analysis was performed using propensity score matching based on logistic regression as the estimation algorithm (nearest-neighbor matching, ratio 1:1, caliper = 0.5), and accounting for hematoma volume as the major determinant of outcome.

We used the Kolmogorov–Smirnov test to determine the distribution of data. Because the data turned out to be non-normally distributed, data were presented as the median and interquartile range (IQR) and then compared between groups using the Mann–Whitney *U-*test. Pearson's χ^2^ or Fisher's exact test was used to compare frequency distributions of categorized variables between dichotomized patient groups.

## Results

### Patient Characteristics

In the MIS center, 74 patients were identified as VKA-related ICH. Thirty patients were excluded due to a hematoma volume <20 ml, five patients due to a GCS of 15 (no reduced level of consciousness), and seven patients due to infratentorial location. Five patients were excluded due to an INR <1.8, 15 patients due early limitation of therapy, two patients due to an arterio-venous malformation in CT angiography, five patients due to a platelet count <100,000/μl, two patients due to open surgery performed as rescue therapy for a large ICH (respective hematoma volumes of 81 ml and 91 ml), with signs of incipient herniation. Two patients were additionally treated with induced hypothermia. Thus, 21 patients were allocated to a drainage insertion procedure.

One hundred and nine patients in the historical control group were identified as having VKA-related hemorrhage. Nineteen patients were excluded due to insufficient imaging, three patients due to open surgery as lifesaving therapy for large ICH (77–105 ml) with signs of incipient herniation, one patient due to hypothermia, two patients due to aborted VKA intake prior to admission, two patients due to infratentorial location, 16 patients due to early limitation of therapy, and 31 patients due to a hemorrhage volume <20 ml. This led to 35 patients being included in the control group.

It is important to note that the median initial hematoma volume differed significantly between groups [control group: 26.4 ml (IQR: 20.1–44.3); MIS group: 57.5 ml (IQR: 35.8–72.2), *p* = 0.001], and the median PHE was significantly larger at admission in the MIS group [39 ml (IQR: 26–71) vs. 23 ml (IQR: 12–39), *p* = 0.01]. On day 7, the median hematoma volume was reduced by 58% to 24 ml in the MIS group (IQR: 17–42) compared to a 16% increase to 31 ml in the control group (IQR: 18–51). The increase in PHE during the first week was less pronounced in the MIS group [−1.7 ml (IQR: −26.8 to 11.9) and 6.2 ml (IQR: −2 to 15.2), *p* = 0.075].

### Propensity Score Matching Groups

Due to significant differences in the initial hematoma volume and PHE volume, propensity score matching was performed, resulting in 16 patients in both groups. Table 1 reveals well-balanced baseline characteristics, indicating a sufficient PS match. The median INR on admission was 2.75 (IQR: 2.3–3.3) in the MIS group and 3.15 (IQR: 2.25–3.83) in the control group (*p* = 0.522). The median time from symptom onset to admission was 4 h (range: 2–8.5 h) in the MIS group and 3.5 h (range: 1.5–7 h) in the control group (*p* = 0.91). The median time from the first CT scan to the initiation of PCC infusion was 27 min (range: 20–50 min) in the MIS group and 36 min (range: 15–70 min) in the control group (*p* = 0.93).

**Table 1:** Baseline characteristics of the propensity score-matched cohort (*n* = 32).

**Characteristics of the propensity score-matched cohort (*n* = 32)**	**Control group; *n* = 16**	**MIS group; *n* = 16**	***p*-value**
Age (IQR) [years]	76.5 (70.5–80.5)	74 (67.25–80)	0.462^#^
Sex (male) [%]	10 (63)	10 (63)	0.999^+^(ex.)
Hypertension (%)	14 (88)	15 (94)	0.999^+^(ex.)
Diabetes mellitus (%)	4 (25)	3 (19)	0.999^+^(ex.)
INR at admission (IQR)	3.15 (2.25–3.83)	2.75 (2.3–3.3)	0.522^#^
Platelet count (IQR) [10e3/μl]	229 (174–308)	206 (176–267)	0.396^#^
PTT (IQR) [s]	42.1 (34.4–50.8)	46.0 (38.3–52.7)	0.624^#^
ICH volume at admission (IQR) [ml]	45.7 (24.2–56.7)	48.4 (28.7–59.6)	0.327^#^
Lobar ICH location (%)	8 (50)	8 (50)	0.999^+^(ex.)
IVH (%)	8 (50)	11 (69)	0.473^+^(ex.)
EVD (%)	6 (26)	7 (44)	0.999^+^(ex.)
mRS at discharge (IQR)	5 (4, 5)	5 (4, 5)	0.949^#^
In-hospital mortality (%)	2 (13)	0 (0)	0.484^+^(ex.)

*MIS, minimally invasive surgery; INR, international normalized ratio; PTT, partial thromboplastin time; EVD, extra-ventricular drain; mRS, modified Rankin Scale; ICH, intracerebral hemorrhage. Data are presented as the median and interquartile range (IQR), or number and percentage (%)*.

+ * χ^2^/Fisher's exact test (ex.) (performed where necessary)*,

# * Wilcoxon rank sum test*.

The location of the ICH was lobar in 50% (8/16) of patients in each group. The median GCS on admission was 14 (IQR: 3–14) in the control group and 13 (IQR: 9–14) in the MIS group (*p* = 0.765). The median NIHSS on admission was 12 (IQR: 6–27) and 15 (IQR: 12–22), respectively (*p* = 0.438).

### Evolution of ICH Volume, PHE Volume, and Midline Shift

ICH volume on admission was 48.4 ml in the MIS group and 45.7 ml in the control group (*p* = 0.327). The median duration from symptom onset to catheter placement was 8 h (range: 6–21 h). In the MIS group, aspiration of a median blood volume of 15 ml (IQR: 10–23) was possible after catheter placement, resulting in a reduction of 11.1 ml in median hematoma volume in the time between scanning at admission and control scanning after catheter positioning.

**Figure 2:**
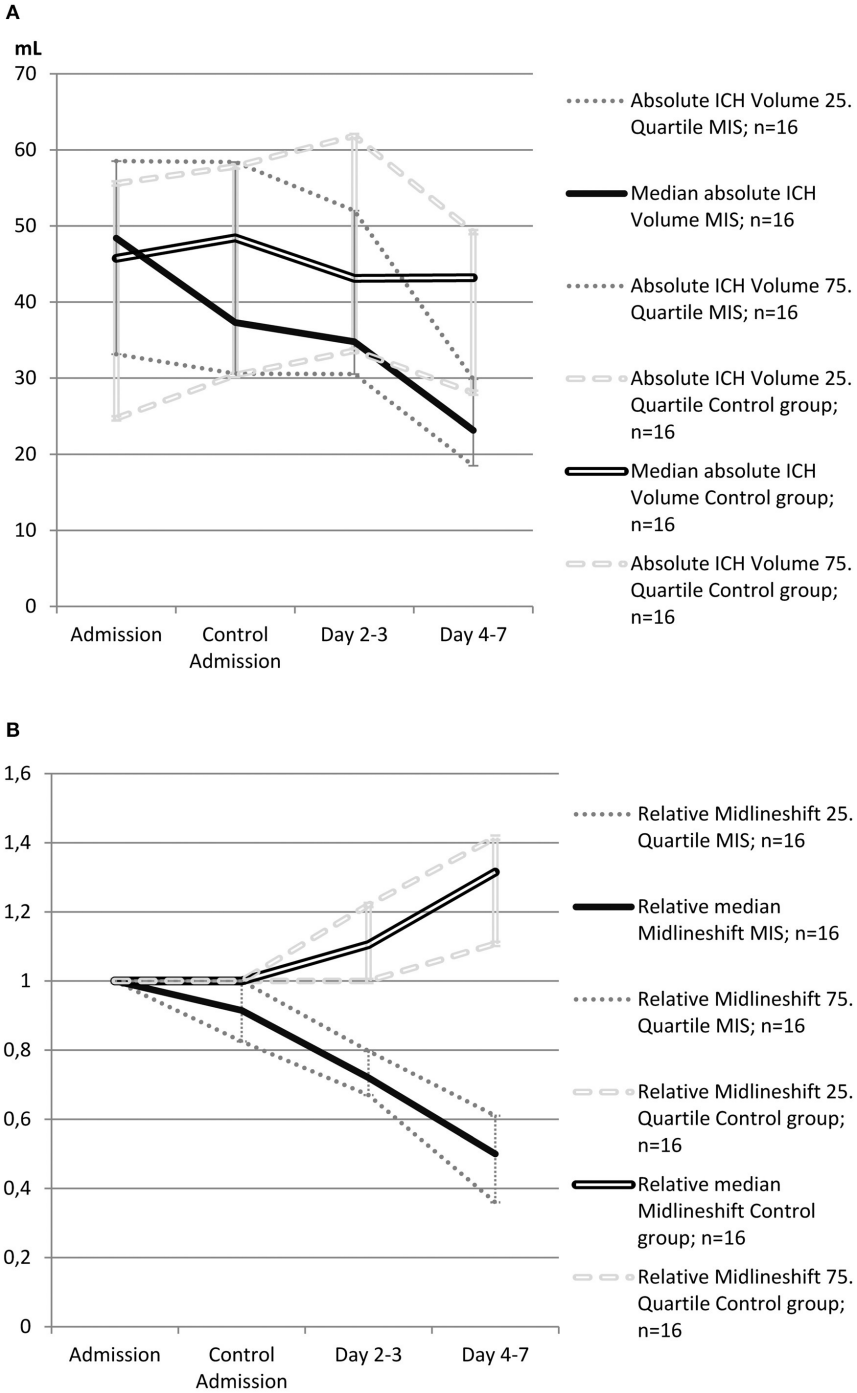
**(A)** Evolution of absolute intracerebral hemorrhage (ICH) volume in patients treated with minimally invasive surgery (MIS) and in control patients from the propensity score-matched cohort (*n* = 32). The single bold line represents MIS patients, and the double line represents control patients. Dotted gray lines represent interquartile ranges. **(B)** Evolution of relative midline shift in patients treated with minimally invasive surgery (MIS) and in control patients from the propensity score-matched cohort (*n* = 32). Midline shift at admission was defined as 1 (100%), and other values were calculated as the ratio of the admission value. The single bold line represents MIS patients, and the double line represents control patients. Dotted gray lines represent interquartile ranges.

Local thrombolysis (5000 IE of urokinase, every 6 h) was started 4 h (range: 3–5 h) after catheter placement and performed for 4 days (IQR: 3–4). ICH volume in MIS patients on day 7 was significantly reduced compared to controls (23.2 ml vs. 43.2 ml, *p* = 0.013, Figure 2A). Therefore, bedside hematoma aspiration and subsequent thrombolysis resulted in a 52%, reduction in hematoma volume, whereas this volume remained almost unchanged for over 7 days in the control group.

PHE volume on admission was 23.8 ml in the control group and was observed to continuously increase to 31 ml by day 7 (an increase of 30%). In the MIS group, the initial PHE volume was slightly higher (33.6 ml, *p* = 0.44) and increased to 42.4 ml by day 7 (an increase of 26%).

The course of the midline shift is shown in Figure 2B. The absolute values of midline shift on admission did not differ significantly between both groups, albeit with a trend toward a higher midline shift on admission in MIS patients (6.5 vs. 3.0 mm, *p* = 0.057). In control patients, the midline shift progressed up to day 7, whereas in the MIS group, the midline shift already began to decrease after catheter aspiration, and continued to decline at all-time points measured. This resulted in a significant reduction in midline shift within the time between admission and day 7, when compared to that of the medical treatment group (*p* < 0.001, see Table 2).

**Table 2:** Evolution of hematoma volume, peri-hemorrhagic edema volume, and midline shift in the propensity score-matched cohort (*n* = 32).

**Characteristics of the propensity score-matched cohort (*n* = 32)**.	**Control group; *n* = 16**	**MIS group; *n* = 16**	***p*-value**
ICH volume at admission (IQR) [ml]	45.7 (24.2–56.7)	48.4 (28.7–59.6)	0.327
ICH volume in control CT scan on day of admission/post-interventional in the MIS group (IQR) [ml]	48.4 (30.0–58.8)	37.3 (30.2–59.5)	0.910
ICH volume, days 2–3 (IQR) [ml]	43.1 (29.9–62.2)	34.8 (30.2–54.0)	0.366
ICH volume, days 4–7 (IQR) [ml]	43.2 (27.5–52.4)	23.2 (15.8–32.3)	0.013
PHE volume at admission (IQR) [ml]	23.8 (12.4–45.5)	33.6 (18.8–50.8)	0.440
PHE volume in control CT scan on day of admission/post-interventional in the MIS group (IQR) [ml]	27.8 (17.8–53.0)	35.5 (28.1–55.7)	0.258
PHE volume days 2–3 (IQR) [ml]	30.7 (15.9–48.0)	39.9 (22.4–69.5)	0.309
PHE volume days 4–7 (IQR) [ml]	31.0 (18.8–53.8)	42.4 (25.0–72.3)	0.274
Midline shift, at admission (IQR) [mM]	3 (0–7)	6.5 (5–10.25)	0.057
Midline shift in control CT scan on day of admission/post-interventional in the MIS group (IQR) [mM]	4.5 (0–7.75)	6 (4.4–10)	0.177
Midline shift, days 2–3 (IQR) [mM]	4.75 (1.25–7.6)	5.25 (3.75–8)	0.777
Midline shift, days 4–7 (IQR) [mM]	5.5 (0.5–9.4)	3 (1.8–5.8)	0.347
Increase in midline shift in the period between admission and day 7 (IQR) [mM]	1 (0–2)	−3.75 (−4.25 to −2)	<0.001

*ICH, intracerebral hemorrhage; PHE, peri-hemorrhagic edema. For relative levels of ICH vs. PHE evolution, volume was defined as 100% in each patient at admission. Data are given as median and interquartile range (IQR) and compared between groups using the Wilcoxon rank sum test*.

### Adverse Events

No symptomatic re-bleeding occurred in the MIS group, and no serious complications related to catheter placement (such as infection) or thrombolysis were observed. Of note, no routine microbiological testing of the catheter tip on removal was performed.

In all MIS patients, blood could be aspirated after initial catheter insertion (median: 15 ml, IQR: 10–23) and the control CT revealed a reduction in hematoma volume in all patients. Furthermore, there was no evidence of asymptomatic or symptomatic hematoma expansion, nor any bleeding along the catheter trajectory on CT. After control CT scanning, the catheter was deemed to be well positioned (i.e., with the fenestrated segment in the core of the hematoma) in 7 (44%) patients and in a sub-optimal position (eccentric hematoma location, but fully engaging the ICH and suitable for urokinase application) in five patients (31%). In 2 patients (12%), the tip of the catheter was outside the hematoma, while the body of the catheter engaged the clot. In these cases, the catheter was retracted a few centimeters, and the CT control revealed an appropriate catheter position for thrombolysis. The catheter had to be replaced in 1 patient (6%) because the catheter perforations were not in contact with the blood clot.

Two patients showed signs of ventriculitis (increases both in CSF cell count and lactate) that was promptly treated with i.v. antibiotics. However, both patients had received an external ventricular catheter for hydrocephalus due to ventricular hematoma expansion. Thus, ventriculitis was likely associated with the external ventricular catheter.

### Functional Outcome at Discharge

Patients were discharged from hospital after a median of 13 days (IQR: 11–19) in the control group and after 14 days (IQR: 10–20) in the MIS group. All patients in the MIS-group and 14 patients in the control group were discharged to a rehabilitation clinic. The mRS score at discharge did not differ between groups [mRS: 5 (4, 5) in both groups, *p* = 0.949, see Table 1].

The median GCS on admission was 13 (IQR: 9–14) in the MIS group and 14 (IQR: 3–14) in the control group (*p* = 0.765). At the time of discharge to the rehabilitation clinic, the GCS in the MIS group had improved in 13 patients (81%), but remained unchanged in 3 (19%) [median GCS: 14 (IQR: 13–15)]. In the control group, the GCS had improved in 7 (44%) patients, remained unchanged in 3 (19%), and deteriorated in 4 (25%) patients. Two patients had died [median GCS: 14 (IQR: 9–14); *p* = 0.85].

Neurological improvement as measured by the NIHSS was observed in 13 patients (81%) in the MIS group, whereas only 6 (38%) patients showed improvement in the control group. As a consequence, the median NIHSS improved from 15 (IQR: 12–22) to 12 (IQR: 8–17) in the MIS group, and deteriorated from 12 (IQR: 6–27) to 15 (IQR: 9–18) in the control group [median change in NIHSS from the time of admission to discharge: −4 (IQR: −7 to −2) and 0.5 (IQR: −12 to 8); *p* = 0.151].

Three patients in the MIS group had a thalamic ICH location. After the MIS procedure, 1 patient with a right-sided thalamic ICH showed an improvement in the NIHSS score, which was 20 at admission and 12 at discharge. The GCS improved from 10 to 14, and the mRS was 4 at discharge. The other two patients with left-sided thalamic ICH remained clinically unchanged after the MIS procedure (NIHSS score: 19 and 22 at admission, and 19 and 20 at discharge, respectively; each patient had a mRS of 5 at discharge).

The in-hospital mortality rate was slightly reduced in MIS patients and did not reach the pre-defined level of statistical significance (Table 1).

## Discussion

Our retrospective analysis allowed us to show that a free-hand bedside catheter approach using urokinase-based thrombolysis is a safe and feasible therapy for patients with VKA-related, large supratentorial ICH. When applied after immediate reversal of anti-coagulation with PCC, this approach resulted in a significant reduction in both hematoma volume and midline shift.

An outcome-modifying treatment approach is yet to be established in ICH patients (2). Hematoma volume is a key prognostic factor for poor outcome (25), with two major pathophysiological mechanisms: first, the ICH leads to mechanical disruption of neurons and glia, where an additional volume-related, space-occupying effect can potentially lead to reduced cerebral blood perfusion, the compression of more distal brain parenchyma, or even herniation (1, 2). Second, the presence of blood induces the activation of detrimental pathways that can subsequently lead to inflammation, peri-hemorrhagic edema, and, in turn, further neuronal damage (27, 28).

Thus, a rapid reduction in hematoma volume would theoretically represent the treatment of choice to improve outcome in ICH patients. However, clinical data from randomized controlled trials, as well as several meta-analyses (3–6), were not able to show any general advantages of craniotomy-related clot removal in ICH patients, compared to those receiving standard medical treatment (2). Most of the meta-analyses included studies that use different surgical techniques (i.e., conventional open surgery vs. minimally invasive approaches) in the intervention group for comparison to best medical treatment, thus limiting interpretation of the results. The failure of conventional surgical management to improve outcome in (neurologically stable) patients with supratentorial ICH has been attributed to a possible traumatic event in the surrounding brain parenchyma caused by the surgical approach, which negates the benefit of hematoma evacuation. The available data on VKA-related ICH are even more limited, in which the two largest randomized controlled studies, STICH I and II (3, 4), only included <10% of patients with VKA-related ICH.

Since hematoma volume remains one of the most important prognostic factors and open surgery could not be shown to improve outcome in ICH patients, minimally invasive procedures are increasingly being developed and have so far shown encouraging results (2). A meta-analysis of 4 RCTs with a total of 2,996 participants (29), and another meta-analysis of 11 studies with a total of 1,717 patients (30) reported a significant reduction in the odds of death or dependency at the final follow-up in patients treated with stereotactic aspiration compared to those who had undergone craniotomy. However, in the MISTIE-III trial (10), the intention-to-treat analysis did not uncover a functional benefit of catheter-based local thrombolysis.

The use of MIS in ICH can be grouped into two broad categories: [1] stereotactic catheter placement and aspiration, followed by thrombolysis to facilitate passive drainage of the hematoma over multiple days, and [2] primarily mechanical, active evacuation, with the goal of removing the hematoma in a single procedure, typically by endoscopic or endoscopic-assisted evacuation. However, such MIS techniques usually require an advanced neurosurgical setup in the operating room, with the need for general anesthesia and specific stereotactic or image-guided procedures.

Furthermore, in most of the MIS trials on ICH, including the largest MISTIE trial (10), <10% of the study patients presented with a VKA-related ICH, further limiting the data on this type of ICH.

In the present study on patients with supratentorial lobar and deep VKA-associated ICH, we applied a free-hand, bedside catheter evacuation procedure that has been previously described for isolated traumatic hematoma, cerebellar hemorrhage (31, 32), and cerebral amyloid angiopathy-related ICH (19). The procedure was performed after immediate reversal of anti-coagulation to an INR <1.3 using PCC, and the catheter could be placed 8 h after symptom onset (range: 6–21 h). We did not observe complications related to catheter placement or subsequent thrombolysis with urokinase, such as re-bleeding or local infection. Blood aspiration was possible in all cases, and catheter position was satisfactory in 81% of patients. The catheter only needed to be replaced in one patient.

By performing this procedure free-hand at the bedside and without intubation or general anesthesia, the delay in time from the ICH diagnosis to surgical intervention is short. The whole procedure takes approximately 15 min to complete, which is markedly lower than the time needed for open surgery or stereotactic catheter positioning. Moreover, there is no need for an operating room with specialized equipment, personnel, or general anesthesia. The latter often results in patients remaining intubated post-operatively, with subsequent adverse effects.

We were able to show that hematoma volume significantly decreased by 50% in MIS patients within the first 4–7 days, while in conservatively treated patients, ICH volume remained almost unchanged. Notably, the 69% clot reduction that was reported in the MISTIE trial could not be achieved in the present study. However, the targeted reduction in hematoma volume to <15 ml in the MISTIE trial—a somewhat ambitious target even in non-anticoagulant-associated ICH patients—cannot simply be transferred to patients with VKA-associated ICH, where the risk of re-bleeding by locally administered urokinase is likely to be higher. Therefore, we chose the more achievable goal of reducing the hematoma by 50%, which may be more suitable in these particular patients. Our findings are essentially in line with previous studies on stereotactic catheter-based evacuation (10).

Interestingly, PHE volume in the MIS group was not significantly reduced in comparison to that of the control group, although a non-significant trend toward a less-pronounced increase in PHE was observed during the first week in the MIS patients when analyzing the total cohort (*p* = 0.075).

Two previous trials have shown a significant reduction in absolute PHE associated with catheter aspiration, followed by local thrombolysis with rtPA or urokinase. In the MISTIE-II trial (thrombolysis with rtPA), edema volume was reduced by 18% in the MIS group compared to a 40% increase in the control group (11), and in CAA-related lobar ICH patients, the same MIS procedure as that used in the present study resulted in a 20% reduction in PHE volume (19).

The lack of an observed effect of MIS on edema evolution in the present study may be explained at least in part by the rather low increase in PHE volume in the control group in relation to the “normal” PHE evolution in conservatively treated patients with spontaneous, non-VKA-related ICH (27, 33). Here, PHE is reported to increase continuously over the initial postictal period, with edema volume almost having doubled by 7–10 days. Given that PHE evolution has been shown to depend, among other things, on thrombin (34), VKA-related ICH *per se* may be associated with reduced PHE evolution. One clinical study has already confirmed that PHE volume is reduced in warfarin-related ICH compared to non-coagulopathic ICH (35).

Experimental data have suggested that the local use of thrombolytic agents may potentiate brain edema formation by thrombin release, due to its pro-inflammatory and neurotoxic properties (36). However, this observation has not been reported in clinical studies using rtPA in spontaneous ICH or intraventricular hemorrhage (37, 38). Moreover, in the present study, we used urokinase as a fibrinolytic agent. There is some evidence that urokinase is at least as efficient as rtPA in local clot lysis, although the well-known neurotoxic and pro-inflammatory effects of rtPA have not been reported for urokinase (26, 39). Thus, based on these observations, it seems unlikely that local thrombolysis with urokinase potentiates edema formation and thereby negates the positive effect of hematoma reduction on edema evolution. Theoretically, the administration of PCC (containing prothrombin) may aggravate brain edema evolution. However, since the timing and dosage of PCC infusion did not differ between groups in the present study, the lack of effect on edema evolution in the MIS group is not likely attributable to PCC administration.

As a consequence of hematoma reduction and the relatively stable PHE in the MIS group, the midline-shift—which serves as an indicator of the lesion's mass effect—decreased immediately after catheter aspiration, and continued to decline over the first week. In the control group, however, hematoma volume remained unchanged while PHE slowly increased, resulting in progressive midline shift.

Based on assessment with the mRS, short-term outcome was similarly poor in both groups. However, the mRS, which is usually used for the estimation of long-term outcome, is unlikely to be sensitive enough to capture moderate differences in clinical course in the acute–subacute phase of patients with large ICH. According to the NIHSS, a trend toward better neurological improvement during the first two postictal weeks was observed in the MIS group (improvement in 81% of patients vs. 44% in the control group), although this did not differ significantly to the control group (*p* = 0.151). Two patients in the control group (13%) died due to space-occupying mass effect and herniation, whereas there were no patient deaths in the MIS group. This is of particular importance, since 69% of MIS patients had intraventricular extension of the hemorrhage compared to 50% of the control patients, and clinical deficit on admission was slightly more severe in the MIS group.

There are several limitations to our study. First, our findings are based on a retrospective analysis of the MIS approach that was performed in a small cohort from a single center and then compared to historical control group data from another center. As mentioned, this inter-center comparison was associated with significant group differences in terms of ICH volume on admission. However, we performed a PS match to control for this difference. As a second major limitation, unlike what was performed in the MISTIE trial, no regular CT scanning was carried out prior to the MIS procedure to ascertain clot stability, nor was a CT performed at pre-specified time points. The lack of these particular measures might therefore impose a possible bias on our analysis of secondary hematoma expansion. According to institutional protocol, a stability CT was only performed in cases of clinical deterioration, in order to minimize the time between admission and the MIS procedure in patients who already presented with reduced consciousness due to the mass effect of the hematoma. Furthermore, we only included VKA-related ICH patients, which limits the generalizability of our data, especially of that relating to direct oral anticoagulant (DOAC)-related ICH. However, as the existing data on VKA-related ICH are limited, our findings provide the first insights into the feasibility and safety of a bedside MIS procedure in these particular patients. Finally, we cannot provide long-term outcome data and our study was not powered for sufficient functional outcome testing.

## Conclusion

We have shown that minimally invasive catheter evacuation with urokinase is feasible and safe when performed free-hand at the bedside in patients with VKA-related ICH. Furthermore, this approach could reduce the mass effect of the hematoma. Bedside MIS might be a promising treatment option for these high-risk patients with larger hematoma volumes, as no additional operating setup is needed. Further studies are required to evaluate the impact of this procedure on functional outcome.

## Data Availability Statement

The raw data supporting the conclusions of this article will be made available by the authors upon reasonable request.

## Ethics Statement

The studies involving human participants were reviewed and approved by Ethik-Kommission der Albert-Ludwigs-Universität Freiburg. Written informed consent for participation was not required for this study in accordance with the national legislation and the institutional requirements.

## Author Contributions

BV, W-DN, SA-S, DS, MS, SL, HL, JK, HH, SS, and JB: acquisition, analysis and interpretation of data for the work, revising the manuscript, and final approval of the version to be published. BV and JB: conception and design of the work and drafting the work. All authors contributed to the article and approved the submitted version.

## Conflict of Interest

The authors declare that the research was conducted in the absence of any commercial or financial relationships that could be construed as a potential conflict of interest.
